# A Pasteur Institute in New York

**Published:** 1886-09-20

**Authors:** 


**A Pasteur Institute in New York.**—Dr. Valentine Mott recently arrived in this city from Paris with a rabbit which had been inoculated by Pasteur, and an institute for the cultivation of the virus of rabies and the inoculation of human subjects has been organized, with Dr. Alexander B. Mott as president. The *Lancet* reports that M. Pasteur has been so favorably impressed by the scientific intelligence of Dr. Valentine Mott that he has for the first time, broken through his rule not to allow his virus to go out of his own hands.—*Gaillard's Medical Journal.*

